# A reliable method for the detection of *BRCA1* and *BRCA2* mutations in fixed tumour tissue utilising multiplex PCR-based targeted next generation sequencing

**DOI:** 10.1186/s12907-015-0004-6

**Published:** 2015-03-24

**Authors:** Gillian Ellison, Shuwen Huang, Hedley Carr, Andrew Wallace, Miika Ahdesmaki, Sanjeev Bhaskar, John Mills

**Affiliations:** Personalised HealthCare and Biomarkers, Innovative Medicines and Early Development, AstraZeneca, Alderley Park, Macclesfield, SK10 4TG UK; Genomic Diagnostics Laboratory, Manchester Centre for Genomic Medicine, Central Manchester University Hospitals NHS Foundation Trust, Saint Mary’s Hospital, Oxford Road, Manchester, M13 9WL UK; Translational Science, Oncology Innovative Medicines Unit, AstraZeneca, Alderley Park, Macclesfield, SK10 4TG UK; R&D Information, AstraZeneca, Alderley Park, Macclesfield, SK10 4TG UK

## Abstract

**Background:**

Germline mutations in *BRCA1* or *BRCA2* lead to a high lifetime probability of developing ovarian or breast cancer. These genes can also be involved in the development of non-hereditary tumours as somatic *BRCA1/2* pathogenic variants are found in some of these cancers. Since patients with somatic *BRCA* pathogenic variants may benefit from treatment with poly ADP ribose polymerase inhibitors, it is important to be able to test for somatic changes in routinely available tumour samples. Such samples are typically formalin-fixed paraffin-embedded (FFPE) tissue, where the extracted DNA tends to be highly fragmented and of limited quantity, making analysis of large genes such as *BRCA1* and *BRCA2* challenging. This is made more difficult as somatic changes may be evident in only part of the sample, due to the presence of normal tissue.

**Methods:**

We examined the feasibility of analysing DNA extracted from FFPE ovarian and breast tumour tissue to identify significant DNA variants in *BRCA1/ BRCA2* using next generation sequencing methods that were sensitive enough to detect low level mutations, multiplexed to reduce the amount of DNA required and had short amplicon design. The utility of two GeneRead DNAseq Targeted Exon Enrichment Panels with different designs targeting only *BRCA1/2* exons, and the Ion AmpliSeq BRCA community panel, followed by library preparation and adaptor ligation using the TruSeq DNA PCR-Free HT Sample Preparation Kit and NGS analysis on the MiSeq were investigated.

**Results:**

Using the GeneRead method, we successfully analysed over 76% of samples, with >95% coverage of *BRCA1/2* coding regions and a mean average read depth of >1000-fold. All mutations identified were confirmed where possible by Sanger sequencing or replication to eliminate the risk of false positive results due to artefacts within FFPE material. Admixture experiments demonstrated that *BRCA1/2* variants could be detected if present in >10% of the sample. A sample subset was evaluated using the Ion AmpliSeq *BRCA* panel, achieving >99% coverage and sufficient read depth for a proportion of the samples.

**Conclusions:**

Detection of *BRCA1/2* variants in fixed tissue is feasible, and could be performed prospectively to facilitate optimum treatment decisions for ovarian or breast cancer patients.

**Electronic supplementary material:**

The online version of this article (doi:10.1186/s12907-015-0004-6) contains supplementary material, which is available to authorized users.

## Background

Mutations in the *BRCA1* and *BRCA2* genes lead to an increased risk of developing breast or ovarian cancer as part of hereditary breast-ovarian cancer syndrome. Women who are heterozygous for a *BRCA1* or *BRCA2* pathogenic variant have up to an 80% risk of developing breast cancer by age 90; and an ovarian cancer risk of about 55% with *BRCA1* mutations and 25% with *BRCA2* mutations [1-4].

Researchers have established that these genes can also be involved in the development of non-hereditary, sporadic tumours, as a proportion of ovarian and breast cancers contain somatic (tumour only) *BRCA1* and *BRCA2* pathogenic variants [5-15]. Patients with germline *BRCA* mutations have been shown to derive a clinical benefit from treatment with the poly ADP ribose polymerase (PARP) inhibitor, olaparib [16]. As patients with tumours that harbour a somatic *BRCA* mutation may also benefit from treatment with PARP inhibitors, it is important to be able to test for *BRCA1 and BRCA2* variants in tumour samples available after routine histopathology assessment and diagnosis.

As the majority of clinical specimens are formalin-fixed paraffin-embedded (FFPE) tissue, the subsequent analysis of DNA extracted from such FFPE tumour samples is challenging. Clinical specimens may be small and often yield a limited amount of low quality DNA, thus constraining the analysis that can be undertaken. Unlike the clinically relevant mutation spectrum of genes currently analysed on FFPE tumour DNA, such as *KRAS* or *EGFR,* where the distribution and number of mutations is small, thousands of clinically relevant variations in *BRCA1* and *BRCA2* have been described, and these are distributed widely throughout multiple, large coding regions and intron-exon boundaries [17]. This poses a significant challenge with respect to the accurate detection, analysis time, characterisation and interpretation of sequence variants in *BRCA1* and *BRCA2*.

Tumour samples are histologically heterogeneous [18,19], and tumour-specific DNA will contain varying proportions of contaminating DNA from normal cells. Consequently, methods for somatic mutation detection have to be able to detect DNA changes that may be present in a low proportion of the total DNA isolated from the sample. Next Generation Sequencing (NGS) methods have the potential to detect variants at low admixture levels due to the clonal nature of the method [20]; conversely, screening both *BRCA* genes using methods such as Sanger DNA sequencing requires a significant quantity of input DNA. NGS methods also offer a way to reduce the amount of input DNA required, as the NGS reactions can be highly multiplexed. NGS therefore offers a potential solution to this challenging type of analysis.

In this study we examined the feasibility of analysing ovarian and breast FFPE tumour tissue for significant variants (pathogenic variants, suspected pathogenic variants and variants of uncertain significance [VUS]) in *BRCA1* and *BRCA2* using pre-developed commercially available multiplex PCR library preparation panels for NGS, which had been designed with short amplicons to accommodate fragmented DNA from FFPE tissue.

## Methods

### Samples

A total of 68 ovarian FFPE tumour samples were analysed; these comprised 64 serous carcinomas, 2 endometrioid adenocarcinomas and 2 NOS (not otherwise specified) carcinomas. All samples were obtained from Asterand (Detroit, MI, USA) where they underwent a haematoxylin and eosin pathology review to confirm the presence of tumour in the samples and estimate tumour content. Thirty breast FFPE tumour samples, of unknown subtype, were obtained from Nottingham University (UK). Limited pathology information on the breast samples was provided by the supplier. These samples were collected with appropriate consents that had been reviewed and approved by appropriate regulatory and ethical authorities (further details can be found at Asterand.com and nuhrise.org/nottingham-health-science-biobank).

Control material used was derived from FFPE human tumour explants of known *BRCA* mutation status (HBCX17 *BRCA2* c.6033_6034del, p.(Ser2012GlnfsTer5); HBCX10 *BRCA2* c.9106C>T, p.(Gln3036Ter)); DNA from unfixed human cell line samples previously characterised by Sanger DNA sequencing (MDA-MB-436 *BRCA1* c. 5277+1G>A (homozygote), Cal51 *BRCA2* c.2957delA, p.(Asn986llefsTer14) (heterozygote), HCC1937 *BRCA1* c.5266dupC p.(Gln1756ProfsTer74) (homozygote) and BT474 *BRCA2* c.9281C>A p.(Ser3094Ter) (heterozygote)); and high molecular weight human genomic DNA (Roche) of unknown *BRCA* status. Cell line admixtures of 50%, 25% and 12.5% were prepared using the aforementioned cell lines to a final concentration of 4 ng/μL. For the 50% admixtures, *BRCA1* and *BRCA*2 mutants were mixed in equal proportions (admix 1: MDA-MB-436/Cal51 and 3: HCC1937/BT474); for the 25% admixtures, the genomic DNA (Roche) was added to an equal volume of an aliquot of the 50% admix 1; and for the 12.5% admixture, an aliquot of admix 3 was diluted 1:4 using the genomic DNA.

DNA was extracted from a single 40 μm thick section of each FFPE sample (ovarian tumour, breast tumour and explants) using the QIAsymphony DSP DNA Mini Kit (Qiagen, Hilden, Germany). The resulting DNA was quantified and assessed for quality by quantitative PCR (qPCR) using the Human Genomic DNA (hgDNA) Quantification and QC Kit (KapaBiosystems, Anachem). The ovarian samples were also quantified using a Nanodrop UV spectrophotometer (ThermoScientific, Wilmington, DE, USA). Breast samples were not quantified in this way as the data was not useful. Cell lines were extracted by Tepnel Pharma Services (Manchester, UK) using a proprietary method and quantified by UV spectrophotometry.

### GeneRead V.1 & V.2 panels

Where the concentration of amplifiable DNA determined by the hgDNA Quantification and QC kit (129 bp premix) was greater than 4 ng/μL, samples were normalised to 4 ng/μL using TE buffer. Multiplex PCRs were set up according to the manufacturer’s instructions. For samples where normalisation to 4 ng/μL was possible, 20 ng (5 μL) of DNA was added to each of the four plexes. Where the concentration of DNA was below 4 ng/μL, 5 μL of DNA was added per plex. PCR amplification conditions were as specified by the manufacturer except for those samples where the input DNA concentration was below 2 ng/μL, in which case the number of PCR cycles was increased from 25 to 30. The four PCR plexes for each sample were pooled and purified using QIAquick PCR purification columns (Qiagen) then 2 μL of purified product was diluted 20x and quantified on a 2200 Tapestation (Agilent Technologies Inc., Santa Clara, CA, USA). After quantification, samples were normalised where possible to 4.2 ng/μL using EB buffer (Qiagen) in preparation for end repair using the TruSeq PCR-Free protocol (Illumina, San Diego, CA, USA).

### Ion Ampliseq BRCA1/2 community panel

Where possible, 10 ng of DNA as measured by qPCR at 129 bp, was added to each of the three plexes. For samples where the concentration was lower than 830 pg/μL, 12 μL of DNA was added (the maximum volume of DNA that could be added to each plex). PCRs were otherwise set up according to the manufacturer’s instructions, with the exception that the number of PCR cycles was increased from the recommended 22 to 25. Immediately after amplification, PCR primers were digested using FuPa reagent (LifeTechnologies, Carlsbad, CA, USA). Successful amplification in each plex was monitored by separating 2 μL of PCR product by 2% agarose gel electrophoresis. For samples where no visible PCR amplification was observed, the reactions were repeated as above but with 30 cycles of PCR. The three PCR plexes were then pooled and 2 μL of the pooled product was diluted 20x and then quantified on a 2200 Tapestation (Agilent). After quantification, samples were normalised where possible to 4.2 ng/μL using EB buffer (Qiagen) in preparation for end repair using the TruSeq PCR-Free protocol (Illumina).

### TruSeq PCR Free library preparation

A 60 μL aliquot of each purified pooled plex (250 ng for those samples at 4.2 ng/μL) was end repaired, purified with AmpureXP beads (Agencourt; Beckman Coulter), adenylated, and adapters ligated according to the manufacturer’s (Illumina) protocol. After adapter ligation, the libraries were cleaned up and size-selected to remove adapter monomers and dimers using GeneRead (Qiagen) size selection columns according to the manufacturer’s protocol. The libraries were then quantified in triplicate using the KAPA Library Quantification Kit (Kapa Biosystems) after initial dilution of aliquots to 1:4000 and 1:8000 in EB buffer (Qiagen).

### Library normalisation and MiSeq NGS analysis

After quantification, each sample-specific library was normalised to 0.5nM by the addition of EB buffer (Qiagen). Samples at lower than 0.5nM concentration were left undiluted. Twenty-four samples per NGS MiSeq run were pooled in equal volumes and then denatured with an equal volume of 0.2 N NaOH; they were then neutralised with an equal volume of 200 mM Tris pH 7.0, giving a library concentration of approximately 125pM. Prior to loading on the MiSeq (Illumina), the pooled libraries were diluted to a final concentration of 12.5pM with chilled HT1 solution (Illumina). A 594 μL aliquot of the pooled libraries in HT1 solution was then combined with 6 μL of a 12.5pM PhiX control library and the 600 μL sample loaded on to a MiSeq V.2 300 cycle reagent cartridge and run on a MiSeq using a 2 × 150 bp paired end configuration.

### Bioinformatic analysis

Analysis of deep targeted data and accurate calling of variants (particularly insertions/deletions) remains an evolving field. A best practice pipeline based on the bcbio-nextgen framework [21] was utilised for the processing and analysis of all data. All raw sequence data were processed from fastq files to variant calls using the tools available through bcbio-nextgen. More specifically, BWA-MEM [22] was chosen as the aligner within bcbio-nextgen, and variant calling was performed using an ensemble calling methodology, which combined individual variant calls produced by FreeBayes [23], GATK Unified Genotyper and GATK Haplotype Caller [24]. These variant callers are most suitable for germline variant analysis, namely for summarising differences between the data and the reference genome. However, as no matched normals were analysed and there are no mature, best practice pipelines for tumour-only analysis, it was decided to utilise these mature best practice germline variant calling pipelines for variant detection here. Data for visualising the differences in the amplicon coverage was extracted from the alignment BAM files by using BEDtools [25]. Furthermore, primer trimming of the alignment BAM files was performed using a custom Python program, which assigns each aligned short read to the amplicon with most overlap to a designed amplicon, and soft clips any bases residing outside the amplicons. This was performed as the reference-matching primers of certain amplicons were diluting the allele frequencies of variants observed within overlapping true amplicons. A second pass of variant calling and coverage estimation was performed on the trimmed alignment files. In all cases variant detection frequencies quoted in this paper represent the outputs from the primer trimmed results unless stated otherwise.

High confidence *BRCA1/2* variants were classified through interrogation of publicly available data. Variants initially classified as pathogenic mutations, likely pathogenic mutations or VUS, were confirmed by either Sanger DNA sequencing and/or repeat NGS starting from the original sample. Where a result could not be confirmed it was regarded as an artefact.

### Sanger DNA sequencing

Where possible high confidence variants were confirmed by Sanger sequencing. PCR primers were designed using the Primer 3 program (http://primer3.ut.ee/) with default settings and the ‘Human’ mispriming library setting selected. Amplicons for Sanger sequencing were designed to be <150 bp in size in order to robustly amplify fragmented DNA with the variant in the central third of the amplicon. PCR primer sequences were checked to ensure that they did not overlie any SNP variants and hence be subject to amplification failure using the NGRL diagnostic SNP check tool (https://secure.ngrl.org.uk/SNPCheck/). Primers were synthesized at 50nM scale and cartridge purified (Sigma-Aldrich). Primer sequences are in Additional file 1.

PCR amplification was carried out using Promega GoTaq® PCR mix with each primer at 500nM concentration under standard conditions with 30 PCR cycles and 55°C annealing. PCR amplifications were all carried out in duplicate alongside a known normal control cell line sample. Successful PCRs were purified prior to sequencing using Agencourt® AmpureXP® beads according to the manufacturer’s protocol. Purified PCR products were Sanger sequenced in both forward and reverse orientations using the same primer sequences used for PCR at 200nM final concentration using BigDye® v3.1 according to manufacturer’s cycling conditions. BigDye® v3.1 sequencing reactions were then purified using Agencourt® CleanSeq® beads according to the manufacturer’s protocol and analysed on an Applied Biosystems 3730xl. Sanger sequencing data was analysed alongside the normal control cell line data for the presence of mutations using Mutation Surveyor® software (SoftGenetics).

## Results

### DNA input

**Figure 1:**
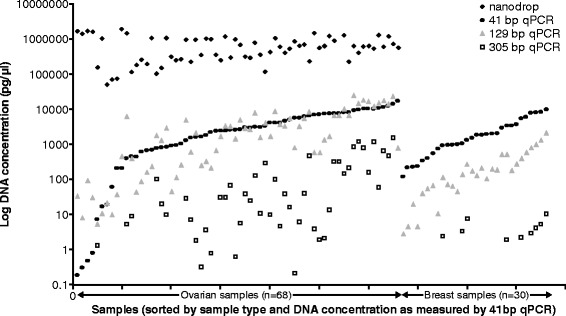
**Comparison of DNA concentration measurements.** DNA quantification data generated by qPCR using 3 different amplicon sizes (41 bp, 129 bp and 305 bp) for all samples studied, and Nanodrop readings for ovarian samples only. The data indicate the level of degradation of DNA in each sample. Ovarian samples appeared to be less degraded than the breast samples as the 41 bp and 129 bp measurements were more similar compared with the marked differential of nearly an order of magnitude observed for the breast samples. Nanodrop readings did not appear to correlate with the qPCR data, generating significantly higher readings as a measure of total DNA rather than amplifiable DNA (ovarian sample data only). Data have been plotted on a log scale to enable a visual comparison.

Although the IonAmpliSeq panel recommended using qPCR for DNA quantification, and the GeneRead panel recommended a spectrophotometric method (Nanodrop), from previous experience of analysing DNA extracted from FFPE material [26], which is typically degraded, we were aware that qPCR was a superior method of estimating amplifiable DNA (DNA of sufficient length to amplify in a PCR reaction) than UV spectrophotometry. The hgDNA Quantification and QC Kit (KapaBiosystems) was therefore selected to perform qPCR and to quantitate the DNA from the FFPE samples. This method used amplicons of 3 different sizes (41 bp, 129 bp and 305 bp) to estimate the quantity and integrity of amplifiable DNA. We found that ovarian tumour samples contained similar quantities of DNA amplifiable by the 41 bp and 129 bp amplicons, whereas breast samples showed a higher value of the 41 bp compared with 129 bp products, suggesting that DNA isolated from breast samples was more degraded. Both sample types showed considerable deterioration in quantities of amplifiable DNA >129 bp, as the 305 bp amplicon generated much lower quantification readings (Figure 1). The ovarian DNA samples were also quantified using a Nanodrop, but these values were considerably higher than qPCR and, in most cases, would have resulted in a substantial overestimation of amplifiable DNA input into the assays (Figure 1). We therefore did not quantify the breast panel in this way to conserve DNA. The 129 bp product was selected to determine the amount of DNA to add into the *BRCA* panel, as it was the closest measure to the mean amplicon size of all methods being evaluated (GeneRead V.1: 155 bp (estimated), V.2: 153 bp, Ion AmpliSeq ~197 bp).

### Comparison of BRCA panel specifications

The *BRCA* panels varied in their specifications, such as the amount of input DNA required; the number of multiplexes; and the gene coverage (Table 1). Before selecting the most appropriate method, however, it was important to test some of these factors empirically to ensure that they fulfilled the claims on typical clinical samples using the equipment available to the laboratory.

**Table 1:** ***BRCA*** **panel specifications**

**Panel name**	Design coverage*	**No. amplicons**	**Mean amplicon size (bp)** ^**+**^	**Amplicon size range (bp)** ^**+**^	**No. primer pools**	**DNA input recommendation**
GeneRead V.1 BRCA1/2	97%	276	155 (104)	(58–125)	4	80 ng total, 20 ng/pool (determined by OD260)
GeneRead V.2 BRCA1/2	100%	237	153 (109)	105-200 (52–159)	4	80 ng total, 20 ng/pool (determined by OD260)
Ion AmpliSeq BRCA1/2	100%	167	197 (145)	126-298 (71–242)	3	30 ng total, 10 ng/pool (determined by qPCR)

*The minimal target regions for 100% design coverage for this assessment was defined as 100% coverage of the *BRCA1* and *BRCA2* CDS (NM_007294.3 and NM_00059.3), respectively, plus at least the 2 bp flanking each coding exon (canonical splice sites). The lengths of these features across the two genes were calculated as 5,680 bp and 10,361 bp for *BRCA1* and *BRCA2*, respectively (total 16,041 bp). Although the designs in many cases covered sequence further into intronic regions, these were not considered in this calculation.

^+^Amplicon size ranges are quoted as length with primer sequences, where known, and length without primer sequences in parentheses.

### GeneRead V.1 (Qiagen)

There were gaps in the coverage of the coding sequences of both *BRCA* genes due to assay design. The theoretical maximum coverage of *BRCA1* and *BRCA*2 (excluding untranslated regions [UTRs], but including 2 bp intron-exon boundaries) was approximately 97.0%. In *BRCA1*, the design coverage was 98.7% with the first 25 bp of coding exon 1 and 42 bp in coding exon 7 omitted as well as some intron-exon boundaries totalling 66 bp of missed sequence. In *BRCA2*, the design coverage was 96.1%, with multiple regions within coding exons 4, 5, 7, 8, 9, 10, 11, 12, 14, 15, 18, 19, 21 and 26 missed as well as multiple exon-intron boundaries totaling 408 bp of missed sequence. With this incomplete coverage, a review of the Breast Cancer Information Core database [27] suggested that around 5% of reported pathogenic variants, if present in the samples, would be missed.

High molecular weight DNA extracted from human cell lines and FFPE explant samples with pathogenic *BRCA* mutations were used as control samples initially, to determine if the GeneRead version1 (V.1) panel functioned adequately. Although the explant samples had been formalin-fixed and embedded in paraffin, the DNA in these samples was not as degraded as the tumour samples, as observed using qPCR. Some admixtures of mutant cell line DNAs with normal control DNA samples were included to obtain some preliminary data on limits of mutation detection. The GeneRead V.1 panel generated adequate results with our protocol, with most samples achieving >100x coverage for 97.0% of the sequence (the theoretical maximum coverage by design) and a mean read depth of >3,900-fold. The exception was one of two explant samples (HBCX17), which performed slightly less well (93.8% coverage at 100x). All the expected mutations were identified in the cell lines and explant samples and none were found in the presumed negative control DNA sample. In admix 4, which was a 12.5% mix of 2 different mutant cell lines in wild type DNA, the *BRCA2* c.9281C > A p.(Ser3094Ter) mutation was not detected automatically by the analysis pipeline, but was present on visual inspection of the data. Since this admixture was derived from a heterozygous mutant sample, in theory the mutant allele would have represented approximately 6% of the total population. These results, presented in Additional files 2 and 3, demonstrated that on first inspection this method was specific, and had the potential to have sufficient limit of detection.

Assay performance was evaluated on typical clinical material that varied in DNA yield, level of degradation and percentages of neoplastic material. Twenty-two percent (22/98) of all samples met the required DNA amount as specified by the GeneRead protocol, and were added at 20 ng per primer pool (4 pools to cover *BRCA1* and *BRCA2*) following the standard protocol (25 PCR cycles). For these samples, the theoretical maximum coverage was achieved at a minimum depth of 100x and with a mean depth of coverage exceeding 4,000x.

As the majority of samples (75/98) yielded less than the recommended input DNA concentration, we evaluated the assay performance on these lower DNA-yielding samples to determine if usable data could be generated. Where the DNA concentration was below half the recommended input (10 ng per pool), the number of PCR cycles was extended to 30 to increase the yield of PCR products. PCR efficiency was monitored initially by agarose gel electrophoresis and then by Tapestation (Agilent).

**Figure 2:**
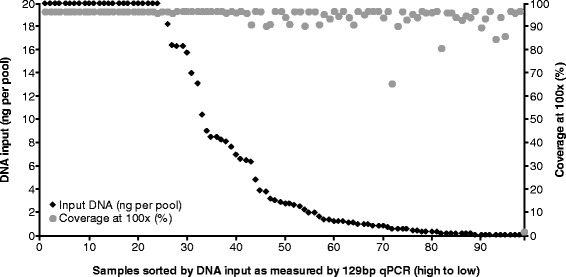
**Percentage coverage at 100x depth compared with DNA input using GeneRead V.1.** Gene coverage is shown over the range of DNA inputs. Approximately 97% coverage (maximum by design) is achieved when the recommended DNA input of 20 ng per plex is used. If less than 20 ng of DNA input per plex is used >95% coverage is still obtained for 70% (52/75) of samples even with as little as 1 ng input DNA per plex.

**Figure 3:**
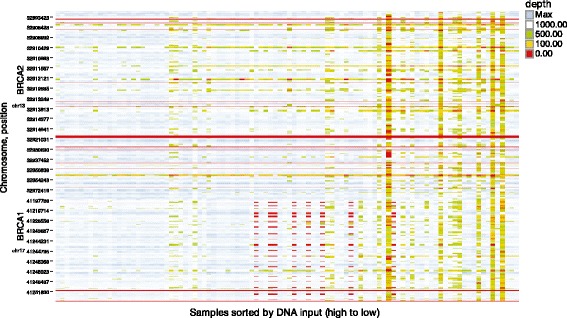
**Heat map of GeneRead V.1 coverage and read depth over the range of DNA input.** As DNA concentration becomes low the read depths tend to become lower. Consistent gaps in the coverage were detected by observing the red continuous horizontal lines.

The majority of samples with low DNA yields produced adequate PCR products and sequencing data without any significant deterioration in coverage or read depth until less than 1 ng of amplifiable DNA was added per primer pool (Figures 2 and 3)*.* Of the 75 samples with less than optimal DNA input, 32 samples still generated the maximum possible coverage of approximately 97%, and a further 20 samples generated a coverage of >95% at a minimum read depth of 100x. The majority of samples gave a mean depth of coverage of >1,000x, which for tumour analysis is important in order to identify low level somatic mutations with confidence.

### Identification of significant variants

Eight samples with pathogenic variants and six samples with VUSs in *BRCA1* and *BRCA2* were identified in the 98 clinical samples tested (Table 2) using the GeneRead V.1 method. These were classified using a diagnostic protocol, which follows professional guidelines adopted in the UK for the interpretation of VUSs [28]. In brief, the protocol integrates publicly available data from peer-reviewed publications, databases and analysis with output from *in silico* tools, and categorises the variant using the 5-class system proposed by the IARC Unclassified Genetic Variants Working Group [29]. Table 2 lists the variants in classes 3, 4 & 5. As we did not have any matched lymphocyte DNA for these patients we were unable to determine if the *BRCA* mutations/variants were germline or somatic events. Twelve of the fourteen variants were confirmed by both repeat NGS and Sanger DNA sequencing. One sample could not be confirmed by Sanger DNA sequencing due to PCR failure, likely due to the poor quality fragmented DNA from the FFPE sample but was successfully confirmed on repeat NGS analysis. Another sample could not be reanalysed by NGS due to insufficient DNA remaining, but was confirmed by Sanger DNA sequencing.

**Table 2:** **High impact variants**

**Sample**	**Sample type**	**High impact variant**	**Class**	**% variant reads**	**NGS replicate confirmation**	**Sanger confirmation**
AZ68^+^	Ovary	*BRCA1* c.1105delG p.(Asp369MetfsTer5)	pathogenic	72	Yes	Yes
AZ75^+^	Ovary	*BRCA1* c.1105delG p.(Asp369MetfsTer5)	pathogenic	68	Yes	Yes
AZ30	Ovary	*BRCA1* c.4675G>A p.(Glu1559Lys)	pathogenic	58	Yes	Yes
AZ28	Ovary	*BRCA1* c.5266dupC p.(Gln1756ProfsTer74)	pathogenic	52	Yes	Yes
AZ23	Ovary	*BRCA2* c.7007+1G>C	pathogenic	79	Yes	Yes
AZ11	Ovary	*BRCA1* c.181T >G p.(Cys61Gly)	pathogenic	84	Yes	No data*
AZ113	Breast	*BRCA1* c.2253_2254delGT p.(Met751IlefsTer10)	pathogenic	52	Yes	Yes
AZ109	Breast	*BRCA1* c.5095C>T p.(Arg1699Trp)	pathogenic	59	Yes	Yes
AZ17	Ovary	*BRCA1* c.2060A>C p.(Gln687Pro)	VUS	90	Yes	Yes
AZ78	Ovary	*BRCA2* c.1408G>C p.(Glu470Gln)	VUS	76	Yes	Yes
AZ72	Ovary	*BRCA2* c.10024G>A p.(Glu3342Lys)	VUS	46	Yes	Yes
AZ39	Ovary	*BRCA2* c.7788delAinsGGGT p.(Gly2596dup)	VUS	58	Yes	Yes
AZ29	Ovary	*BRCA2* c.9302 T>C p.(Leu3101Pro)	VUS	82	No data	Yes
AZ10	Ovary	*BRCA2* c.10095delinsGAATTATATCT p.(Ser3366AsnfsTer4)	VUS	100	Yes	Yes

These variants were confirmed by repeat NGS and validated by Sanger DNA sequencing. One sample* could not be confirmed by Sanger DNA sequencing due to PCR failure, possibly due to the poor quality of the FFPE sample. Another sample was not reanalysed by NGS, but was confirmed by Sanger DNA sequencing. ^+^Samples AZ68 and AZ75 are thought to be separate samples from the same tumour block as they were sourced from the same supplier at the same time and have exactly the same variant calls (including coding SNPs).

**Table 3:** **Detection of variants in decreasing proportions of the total sample using GeneRead V.1**

**Sample**	**Variant**	**Dilution**	**% coverage @100x**	Expected variant allele proportion*	**Observed variant allele proportion**	**Variant reads/total reads**
AZ10	*BRCA2 c.10095delinsGAATTATATCT p.(Ser3366AsnfsTer4)*	1/2	96.9%	~46%	48%	2107/4405
		1/4	97.0%	~23%	20%	1438/7185
		1/8	97.0%	~11%	9%	747/7871
		1/16	96.5%	~5%	4%	322/7591
AZ17	B*RCA1 c.2060A>C p.(Gln687Pro)*	1/2	97.0%	~45%	72.2%	5094/7051
		1/4	97.0%	~23%	55.5%	3428/6174
		1/8	97.0%	~11%	33.6%	2220/6610
		1/16	97.0%	~5.5%	19.1%	1056/5523
AZ23	*BRCA2 c.7007+1G>C*	1/2	96.9%	~40%	67%	2645/3949
		1/4	97.0%	~20%	51%	3235/6340
		1/8	97.0%	~10%	35.1%	3264/9287
		1/16	97.0%	~5%	20.2%	1351/6700

*Expected frequencies were based on results from a previous run where the samples were undiluted.

Seven additional significant variants were identified that could not be replicated or confirmed using an alternative method; these were considered to be PCR artefacts, which are known to occur when analysing DNA extracted from FFPE tissue [26]. The majority of these were in poor quality, low input DNA samples with lower read depth and obvious background sequencing noise (potentially an artefact of PCR from very limiting copies of amplifiable DNA). Repeat analysis by NGS or Sanger DNA sequencing enabled us to readily distinguish true positives from artefacts, as artefacts did not replicate.

### GeneRead V.1 extended evaluation

#### Input DNA

As we were unable to isolate DNA at the recommended input concentration for a high proportion of FFPE tumour samples, we examined the effect of decreasing input DNA on test performance and ability to detect a variant. A FFPE tumour sample containing the *BRCA2* c.10095delinsGAATTATATCT p.(Ser3366AsnfsTer4) variant was processed with a series of input DNA amounts from the recommended 80 ng down to 2.5 ng DNA input total (20 ng down to 0.6 ng DNA input per primer pool). This range of inputs represented 90% of our sample set. This sample series was included as part of a standard analysis batch of 24 samples. The *BRCA2* variant was still detected using a DNA input/pool of 0.6 ng when run in a standard batch of 24 samples (Additional file 4). The percentage of variant reads/allele frequency remained consistent over all the DNA inputs tested and the overall percentage coverage at 100x depth was unaffected by the range of input DNA quantities used. An analysis of four single-nucleotide polymorphisms (SNPs) present in this sample also showed the same consistency over the range of DNA inputs (Additional file 5). This implies that if DNA integrity is good, it is possible to generate reliable results with much lower input DNA amounts than the recommended amount, making analysis possible even in FFPE tumour samples that yield low DNA amounts.

#### Limit of variant detection in a background of DNA

As none of the tumour samples were found to contain a validated low frequency variant, we used a series of decreasing variant content admixtures as a model system to assess the ability of the method to detect variants at low proportions as could occur in a heterogeneous tumour. Three FFPE tumour DNA samples known to harbour *BRCA* pathogenic mutations were mixed with a single background non-mutant FFPE tumour DNA to make 1/2, 1/4, 1/8 and 1/16 admixtures. The total amplifiable input DNA remained constant at 80 ng per test (20 ng per plex) *(Admix set 1: BRCA2 c.10095delinsGAATTATATCT p.(Ser3366AsnfsTer4); Admix set 2: BRCA1 c.2060A>C p.(Gln687Pro); Admix set 3: BRCA2 c.7007+1G>C*). All the variants were clearly detected in the lowest 1/16 admixture (Table 3). Although the relative numbers of reads with the variants declined with the lower admixtures, the actual proportion was not always as predicted and may indicate that the measure of amplifiable DNA at 129 bp qPCR is not fully reflecting the numbers of amplifiable copies of each *BRCA* locus in each sample. Nevertheless, *BRCA* mutations could be readily detected if present in >10% of a sample DNA, which should enable the detection of low level mutations commonly found in tumour material. If the level of neoplastic cells is lower, macro or microdissection to enrich for the proportion of tumour cells may be necessary to achieve the required limit of detection although we did not assess this in this study.

#### Reproducibility of analyses

**Figure 4:**
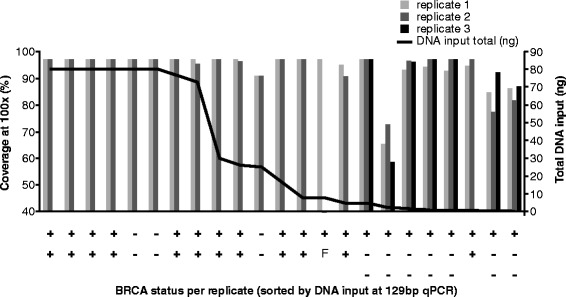
**GeneRead V.1 reproducibility of analyses.** Coverage at 100x depth was reproducible at the recommended DNA input, but as the DNA amount fell below about 1 ng, data were less reproducible. At lower DNA input amounts, *BRCA* status was less reproducible. ++ positive in all replicates (verified *BRCA* positive); −− negative in all replicates (negative); +−− positive in first analysis, negative in replicates (artefact - considered negative); +F positive in 1 replicate, but no NGS result in the replicate.

To evaluate reproducibility, tumour samples that were found to contain a significant mutation were replicated in at least duplicate. In addition, 3 samples that did not contain significant *BRCA* variants were chosen at random, but with sufficient DNA to perform additional analysis, were also replicated. When the DNA input was at the recommended amount the method was highly reproducible, but when the DNA input decreased below 1 ng per test, reproducibility deteriorated. Coverage was not as consistent and non-reproducible artefacts were more likely to occur in the lower DNA-yielding samples. Samples with artefacts were typically evaluated in triplicate to verify the result (Figure 4).

#### GeneRead V.2 panel

As the GeneRead V.2 panel design was not available at the start of the study, it was only possible to conduct a limited evaluation. Twelve FFPE DNA samples from ovarian tumours that had previously been analysed with the GeneRead V.1 panel were re-analysed with the modified (improved coverage) V.2 panel. The theoretical maximum coverage of *BRCA1* and *BRCA*2 (excluding UTRs) was 100%, with no coverage gaps in either gene, and all exon-intron boundaries covered. Eight of the twelve samples selected were analysed using a total DNA input of 80 ng at 129 bp (20 ng of DNA per plex). The remaining four samples were of poorer quality and input DNA ranged between 34 ng - 0.4 ng total input DNA at 129 bp. All twelve samples analysed with the V.2 panel had previously yielded good coverage with the V.1 panel at or very near to the maximum achievable coverage (97%).

One of the twelve samples (AZ68) was an ovarian cancer sample where the pathogenic mutation *BRCA1* c.1105delG p.(Asp369MetfsTer5) had been identified and confirmed. This was also identified on the V.2 panel analysis. No additional mutations were identified using the V.2 panel.

**Figure 5:**
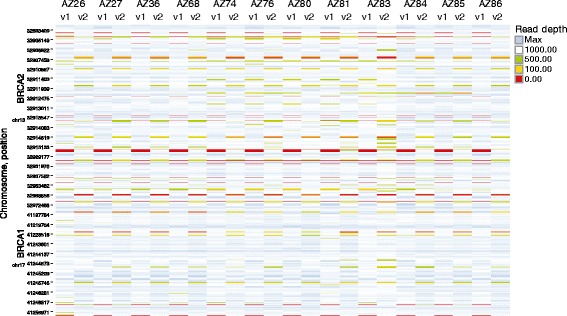
**Comparison of GeneRead panels versions 1 and 2.** Some areas of *BRCA1* and *BRCA2* that had not previously been encompassed in the V.1 design were now covered, but other amplicons from regions previously satisfactorily covered on V.1 failed in the V.2 design.

The overall coverage at 100x minimum depth of the V.2 panel was marginally superior to that obtained with the V.1 panel (Additional file 6). Inspection of the data revealed that although some areas of *BRCA1* and *BRCA2* that had not previously been encompassed in the design were now covered, other amplicons from regions previously satisfactorily covered on V.1 were now failing. Although coverage by design should have been 100%, the maximum achieved in this small evaluation was 98.9% (Figure 5). The V.2 panel would benefit from further optimisation to achieve 100% coverage. It should be noted that V.1 PCR reagents were used in this small evaluation and use of V.2 mastermix may improve the performance but was not tested.

#### Ion AmpliSeq panel

**Figure 6:**
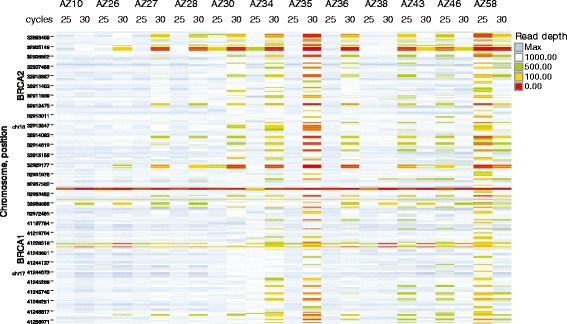
**Gene coverage and read depth using the Ion Ampliseq BRCA1/2 panel; effect of 25 and 30 PCR cycles.** Depth of coverage was generally better at 25 cycles for the majority of samples compared with 30 cycles. Pool 3 products were responsible for the majority of low coverage regions.

A small evaluation of the Ion AmpliSeq panel was conducted, although modification of the protocol was required in order to conduct the analysis on the MiSeq instrument. Twelve FFPE DNA samples from ovarian tumours that had previously been analysed with the GeneRead V.1 panel were analysed with the Ion AmpliSeq panel. Eight samples were added to the assay at the recommended DNA input (30 ng) and 4 samples were added at just below the recommended DNA input. For optimisation purposes, one set of 12 samples was processed using 25 PCR cycles and a second set was processed using 30 PCR cycles as preliminary results had shown some poorly performing samples. Attainment of 100x minimum coverage with the amplicons was variable in this evaluation, including those samples where 30 ng at 129 bp of input DNA was available. Data generated from 25 PCR cycles generally gave better coverage and read depth for the majority of samples but not in all cases (Figure 6 and Additional file 7). The majority of the poorly performing amplicons were in primer pool 3. We investigated the possibility of whether this could be due to primers, PCR amplicon size or GC content. However, the average size of the primer pool 3 products was only about 5 base pairs longer than in other pools, which we did not think should have had such a notable effect, and the GC content, length of primers and numbers of primers were almost identical in all 3 pools.

A table summarising which samples were analysed using which method can be found in the Additional file 8. NGS data has been deposited in The European Nucleotide Archive (www.ebi.ac.uk/ena)*:* submission reference number PRJEB8503.

## Discussion

*BRCA1* and *BRCA2* germline variant screening using Sanger DNA sequencing or NGS is well established in clinical practice and is used primarily for the determination of hereditary breast and ovarian cancer risk [30-33]. The screening methods used are optimised for good quality, high molecular weight input DNA of high yield, usually extracted from blood. These methods do not readily translate to the analysis of FFPE tumour material, where the extracted DNA is typically of poor quality, highly fragmented and of low yield [26]. In addition, Sanger DNA sequencing methods may not be sensitive enough to detect low level somatic changes and are more expensive and difficult to scale for high throughput applications than NGS assays [20]. With the advent of treatment-focused *BRCA* testing and the potential of patients with somatic as well as germline *BRCA* pathogenic or likely pathogenic variants in ovarian tumours to benefit from PARP inhibitor therapy [16], there is an increasing clinical need for routine *BRCA* screening of FFPE tumour DNA. Designing and clinically validating a NGS *BRCA* assay for use on FFPE tissue could take considerable time, effort and cost. The use of pre-developed *BRCA* panels allowed us to rapidly establish a protocol for *BRCA* screening in DNA extracted from FFPE breast and ovarian tumour tissue, and to ascertain the feasibility of routine *BRCA* tumour testing.

To be of clinical utility, any method needs to generate usable results on as many typical FFPE samples as possible, often with low yields of DNA. During this investigation we evaluated *BRCA* panel performances on a range of DNA concentrations and sample quality, as measured by qPCR over 3 amplicon sizes (41 bp, 129 bp and 305 bp). The GeneRead *BRCA* panels required a DNA input of 80 ng in total (20 ng per primer pool or plex) and the Ion AmpliSeq *BRCA* panel an input of 30 ng (10 ng per primer pool). The qPCR reading at 129 bp was used to estimate DNA input as this was closest in size to the range of amplicons in the panels. For routine screening, it is likely we would only run a single qPCR assay with a product size similar to the panel product sizes for DNA estimation in order to conserve DNA and reduce overall turnaround time. For this evaluation, however, it was useful to have more information on the quality and quantity of DNA to allow us to develop the process. We found qPCR was a reliable method for measuring the amount of input DNA to use, and for predicting downstream assay success. When sufficient DNA input as measured by the 129 bp amplicon was added to the *BRCA* tests, the assays performed as expected with maximum achievable coverage and sufficient read depth.

Results generated using the GeneRead V.1 panel demonstrated that close to the theoretical maximum coverage was achievable. This included those samples with as little as 1 ng of amplifiable DNA per primer pool. An average read depth of >1,000x was also attained for the majority of samples. The GeneRead V.2 panel improved the coverage, but still did not achieve its maximum theoretical coverage of 100%. The Ion AmpliSeq assay generated >99% coverage for some samples, but read depth and coverage deteriorated rapidly in other samples, even though the DNA input was sufficient. This deterioration was a particular issue with one of the three primer pools (primer pool 3), with the other two pools appearing to perform better across all samples. We were unable to determine the reason for this; it did not appear to be related to amplicon length, GC content, primer length or number of primers per pool. Perhaps a longer qPCR assay more closely representing the amplicon size, which was slightly longer than the GeneRead panel amplicons, would be a better predictor of starting DNA input, but this was not investigated further. For this reason we did not continue to analyse all the available samples using the Ion AmpliSeq panel after our initial evaluation of 24 samples as we predicted a higher failure rate for the remaining sub-optimal samples. We also did not have access to an Ion PGM or Ion Proton system and ancillary devices to optimise the method on the recommended instrument.

We were able to detect significant variants using both the GeneRead and Ion AmpliSeq panels. All significant variants found in an initial analysis were subject to repeat analysis starting from the original DNA extraction to distinguish true positives from artefacts, as have been described when analysing FFPE DNA using Sanger DNA sequencing. We found a small number of variants that could not be replicated by NGS. As with Sanger DNA sequencing, NGS is also affected by this underlying FFPE DNA quality issue, possibly caused through DNA damage due to deamination and cross-linking during formalin fixation. This problem can be overcome by repeat analysis starting from the original DNA, as the artefacts are generally random in distribution [26]. Artefactual errors were common in poorer quality DNA samples with low input DNA amounts that typically had a higher overall level of background noise, although there were exceptions. These variants were also analysed by Sanger DNA sequencing as a validation of the NGS method. In this study, all the reproducible NGS *BRCA* significant variants were confirmed using Sanger DNA sequencing, with a single exception. This exception was due to limiting amounts of DNA and problems developing a Sanger DNA sequencing assay that would work on the highly degraded DNA, rather than non-detection of the specific variant.

We performed a very limited evaluation of the potential false negative rate (i.e. *BRCA* mutations not detected by the NGS assay). Full gene screens of *BRCA* 1 and *BRCA2* using a comparator method such as Sanger DNA sequencing would not have been practical due to limitations such as the time required to develop an assay for DNA extracted from FFPE tumour tissue, and the amount of DNA that would have been required to carry out complete screens of both *BRCA1* and *BRCA*2. However, all the known control samples with known *BRCA* pathogenic variants were correctly identified (4 cell lines, 2 explants) and the comparison of the different panels did not detect any additional mutations.

We detected 7 different pathogenic variants (in 8 samples) and 6 VUSs in *BRCA1* and *BRCA2* in our panel of ovarian and breast samples. As we did not have access to matched blood samples we were unable to determine if any of these were somatic changes. There was no indication in the allele frequencies in any of the tumours to suggest a low level somatic variant although this does not rule out the possibility that these samples may have a somatic variant at high level and be indistinguishable from a typical germline variant in the tumour.

Further evaluation experiments were conducted with the V.1 panel as this performed better on our samples, to determine if the method was potentially able to detect the low level variants one would predict to be seen in tumour samples with somatic mutations with low neoplastic cell content. By admixing a FFPE sample containing a variant with a FFPE sample containing no mutant, we were able to determine that an allele frequency of about 5% was still detectable when the mean read depth was >1,000x. Although we were aware of the variants we were analysing, it seems reasonable to assume that an allele frequency of down to 10% could be routinely detected.

GeneRead V.1 panel variability was also examined on a small number of samples, including all those with *BRCA* significant variants and 3 without significant variants. When the optimal amount of DNA was added to each plex (20 ng) the data were highly reproducible and continued to generate comparable coverage statistics until the DNA concentration fell below 1 ng.

Although the bioinformatics pipeline used, coupled with visual inspection of the data, allowed us to evaluate the results, it was not optimised or validated for routine screening for *BRCA* mutations in tissue samples. Further work would be required to develop a suitably rapid and validated pipeline for routine analysis of such samples.

While the methods used were capable of effectively detecting point mutations, it is important to note that they have not been designed to detect large rearrangements (genomic insertions or deletions). A significant proportion of pathogenic *BRCA1* and *BRCA2* germline mutations comprise large rearrangements in many populations [34,35], and a method suitable for use on FFPE tissue would still be required to detect this class of mutation. Multiplex ligation-dependent probe amplification (MLPA) is commonly used in diagnostics to detect large gene rearrangements in *BRCA1* and *BRCA2.* Although there is no reason *per se* why MLPA could not be used to detect large rearrangements in FFPE tissue, there are considerable challenges. In particular, any MLPA data analysis method would need to be able to cope with genomic instability in the tumour genome that may affect the control probes used for data normalisation, as well as being able to detect rearrangements present at low level as somatic mutations.

The multiplex PCR panels described in this study have relatively low amplicon tiling (low levels of overlapping amplicons). This low level tiling, although efficient in terms of reducing the complexity of the multiplex primer pools, increases the risk that specific amplicons will fail to amplify due to an unknown variant (e.g. rare or private germline variants) beneath the footprint of one of the primers. Such events will lead to gaps in the read coverage and thus increase the risk of false negative results. As panel designs mature it would be interesting to assess the effects of greater amplicon redundancy, for example, aiming for coverage of every target base with at least two amplicons (2x tiling) with different primer positions.

As the *BRCA* genes are tumour suppressors, two events are required to completely knock out gene function. In cases of hereditary *BRCA* this second hit is typically through loss of heterozygosity [36]. In tumour samples both hits should be present, be they in *BRCA*-mutated carriers or non-carriers if the tumour has arisen due to *BRCA* loss of function. To identify tumours with *BRCA1* and *BRCA2* inactivation that may have the potential to respond to PARP inhibition, multiple technologies will be needed to detect the various mechanisms of gene inactivation.

## Conclusions

In conclusion, we have shown that mutation analysis of *BRCA1* and *BRCA2* is feasible in DNA extracted from FFPE tissue using an NGS approach. However, at present this strategy should not be used as a substitute for comprehensive germline *BRCA* analysis in patients at high risk of having a pathogenic *BRCA1/2* variant, but could be used to identify individuals with somatic only *BRCA1/2* variants who may potentially benefit from PARP inhibition therapy.

In addition, best practice guidelines for the analysis of FFPE tumour [37], germline *BRCA* analysis [38] and assay validation [39] should be followed, such as ensuring significant findings can be replicated in order to ensure the results are reliable before adopting the practice into a clinical diagnostics laboratory.

## Additional files

Additional file 1:
**Sanger DNA sequencing PCR primers.** Primer sequences used to confirm results from BRCA postive samples. Additional file 2:
**GeneRead V.1 panel coverage on control cell lines and tumour explant samples.** All unfixed control samples generated a coverage of 97%, the theoretical maximum by design. One of the fixed explant samples almost achieved maximum coverage while the other was slightly below, possibly due to lower DNA quality in these fixed samples. Additional file 3:
**GeneRead V.1 panel read depth on control cell lines and tumour explant samples.** A mean read depth of >3,900 was obtained for all samples. Regions of lower coverage are apparent in AZ01 (column 6, fixed explant) by the red and green horizontal lines not present in the other samples. The thick red bar near the centre of the heatmap indicates a region in the *BRCA2* CDS not covered in the panel design. Additional file 4:
**Effect on coverage and ability to detect variants compared with DNA input.** The coverage and percentage reads remained consistent as DNA input was reduced. The *BRCA2* c.10095delinsGAATTATATCT p.(Ser3366AsnfsTer4) variant was still detectable at 2.5 ng total DNA input or 0.6 ng DNA input per primer pool. Additional file 5:
**Consistency of percentage variant reads of SNPs.** The figure shows the effect on the percentage of variant reads relative to DNA input. The percentage reads remain consistent across the four SNPs in the regions of interest. The percentage read for rs1799950 is lower, but consistently so. Additional file 6:
**Comparison of GeneRead V.1 and V.2 coverage at 100x minimum depth and mean read depth.** An overall improvement in coverage and depth was observed with V.2, although the maximum coverage by design (100%) was not achieved. Additional file 7:
**Coverage at 100x minimum read depth and effect on coverage at 25 and 30 PCR cycles.** Coverage was better at 25 cycles for the majority of samples compared with 30 cycles. Additional file 8:
**Summary of samples used in each method.**

## Abbreviations

FFPE: Formalin-fixed paraffin-embedded
hgDNA: Human genomic DNA
MLPA: Multiplex ligation-dependent probe amplification
NGS: Next generation sequencing
PARP: Poly ADP ribose polymerase
qPCR: Quantitative polymerase chain reaction
SNP: Single-nucleotide polymorphism
UTR: Untranslated region
VUS: Variant of uncertain significance
