# Supplementary material for: A reliable method for the detection of BRCA1 and BRCA2 mutations in fixed tumour tissue utilising multiplex PCR-based targeted next generation sequencing
Source: BMC Clin Pathol. 2015 Mar 24;15:5. doi: 10.1186/s12907-015-0004-6 (PMC4391122; doi:10.1186/s12907-015-0004-6)
Supplement: Additional file 1: — Sanger DNA sequencing PCR primers. Primer sequences used to confirm results from BRCA postive samples. [file 12907_2015_4_MOESM1_ESM.doc]

**Additional file 1: Sanger DNA sequencing PCR primers.** Primer sequences used to confirm results from BRCA mutation positive samples.

| **Case** | **High Impact Variant** | **F-primer** | **R-primer** |
| --- | --- | --- | --- |
| AZ10 | BRCA2 c.10095delinsGAATTATATCT p.(Ser3366AsnfsTer4) | AGCTGACGAAGAACTTGCAT | GTACTGGCCTGGGAACTCTC |
| AZ11 | BRCA1 c.181T>G p.(Cys61Gly) | N/A | N/A |
| AZ17 | BRCA1 c.2060A>C p.(Gln687Pro) | CAGTCAGGCACAGCAGAAAC | TTCTTCTCTTGGAAGGCTAGGA |
| AZ23 | BRCA2 c.7007+1G>C | CAGTAACATGGATATTCTCTTAGAT | ACGAGACTTTTCTCATACTGTATTA |
| AZ28 | BRCA1 c.5266dupC p.(Gln1756ProfsTer74) | AGCTTCTCTTTCTCTTATCCTGATG | TGCAAAGGGGAGTGGAATAC |
| AZ29 | BRCA2 c.9302T>C p.(Leu3101Pro) | AACACATCTATAATAACATTCTTTTC | ATTTGGATTCTGGTCGCCAC |
| AZ30 | BRCA1 c.4675G>A p.(Glu1559Lys) | AATTGGTGGCGATGGTTTTC | AACCAGAATATCTTTATGTAGGA |
| AZ39 | BRCA2 c.7788delAinsGGGT p.(Gly2596dup) | TGCAGAGTCTTTTCAGTTTCACA | AACACACAATCTTTTTGCATAGA |
| AZ68 | BRCA1 c.1105delG p.(Asp369MetfsTer5) | AATGCTGATCCCCTGTGTGA | TCACTTCTGGAAAACCACTCA |
| AZ72 | BRCA2 c.10024G>A p.(Glu3342Lys) | TTTCAGCCACCAAGGAGTTG | TCTTCTGAACTGGTGGGAGC |
| AZ75 | BRCA1 c.1105delG p.(Asp369MetfsTer5) | AATGCTGATCCCCTGTGTGA | TCACTTCTGGAAAACCACTCA |
| AZ78 | BRCA2 c.1408G>C p.(Glu470Gln) | TGCCACGTATTTCTAGCC | ACCCTGAAATGAAGAAGCCAC |
| AZ109 | BRCA1 c.5095C>T p.(Arg1699Trp) | CAAGGAATTGGTTTCAGATGATGA | GTGCTCCCCAAAAGCATAAA |
| AZ113 | BRCA1 c.2253_2254delGT p.(Met751IlefsTer10) | TTGTCAATCCTAGCCTTCCAAGA | CCTGAGTGCCATAATCAGTACCA |
