# Supplementary material for: A reliable method for the detection of BRCA1 and BRCA2 mutations in fixed tumour tissue utilising multiplex PCR-based targeted next generation sequencing
Source: BMC Clin Pathol. 2015 Mar 24;15:5. doi: 10.1186/s12907-015-0004-6 (PMC4391122; doi:10.1186/s12907-015-0004-6)
Supplement: Additional file 2: — GeneRead V.1 panel coverage on control cell lines and tumour explant samples. All unfixed control samples generated a coverage of 97%, the theoretical maximum by design. One of the fixed explant samples almost achieved maximum coverage while the other was slightly below, possibly due to lower DNA quality in these fixed samples. [file 12907_2015_4_MOESM2_ESM.doc]

**Additional file 2: GeneRead V.1 panel coverage on control cell lines and tumour explant samples. All unfixed control samples generated a coverage of 97%, the theoretical maximum by design. One of the fixed explant samples almost achieved maximum coverage while the other was slightly below, possibly due to lower DNA quality in these fixed samples.**

| **Sample name** | **Total Input DNA  (ng at 129bp)** | **Cycles** | **% coverage** | **Mean read depth** | **High impact variants** | **Level (% variant reads and count of variant over total reads)** |
| --- | --- | --- | --- | --- | --- | --- |
| Wild type control (AZ03) | 80 | 25 | 97.0% | 4815 | None |  |
| Admix 1 50% each cell line (MDA MB 436, Cal51) (AZ13) | 80 | 25 | 97.0% | 3904 | BRCA1 c.5277+1G>A; [splice]  BRCA2 c.2957delA p.(Asn986IlefsTer5) | 20.4% (197/966), 39.3% (1214/3086) |
| Admix 2 25% each cell line in wild type DNA  (MDA MB 436, Cal51) (AZ15) | 80 | 25 | 97.0% | 4543 | BRCA1 c.5277+1G>A; [splice]  BRCA2 c.2957delA p.(Asn986IlefsTer5) | 9% (96/1119)  19.4% (677/3495) |
| Admix 3 50% each cell line (HCC1937, BT474) (AZ16) | 80 | 25 | 97.0% | 4485 | BRCA1 c.5266dupC p.(Gln1756ProfsTer74);  BRCA2 c.9281C>A p.(Ser3094Ter) | 59.1% (686/1161), 21.8% (256/1173) |
| Admix4 12.5% each cell line in wild type DNA  (HCC1937, BT474) (AZ04) | 80 | 25 | 97.0% | 4451 | BRCA1 c.5266dupC p.(Gln1756ProfsTer74);  BRCA2 c.9281C>A p.(Ser3094Ter) | 14.6% (70/480),  4% (46/1048) |
| HBCX17 (AZ01) | 80 | 25 | 93.8% | 4798 | BRCA2 c.9106C>T p.(Gln3036Ter) | 99.6% (967/971) |
| HBCX10 (AZ14) | 80 | 25 | 96.9% | 6452 | BRCA2 c.6033_6034delTT p.(Ser2012GlnfsTer5) | 99.8% (8287/8306) |
