# Supplementary figures and images for: A reliable method for the detection of BRCA1 and BRCA2 mutations in fixed tumour tissue utilising multiplex PCR-based targeted next generation sequencing
Source: BMC Clin Pathol. 2015 Mar 24;15:5. doi: 10.1186/s12907-015-0004-6 (PMC4391122; doi:10.1186/s12907-015-0004-6)

Additional File 3

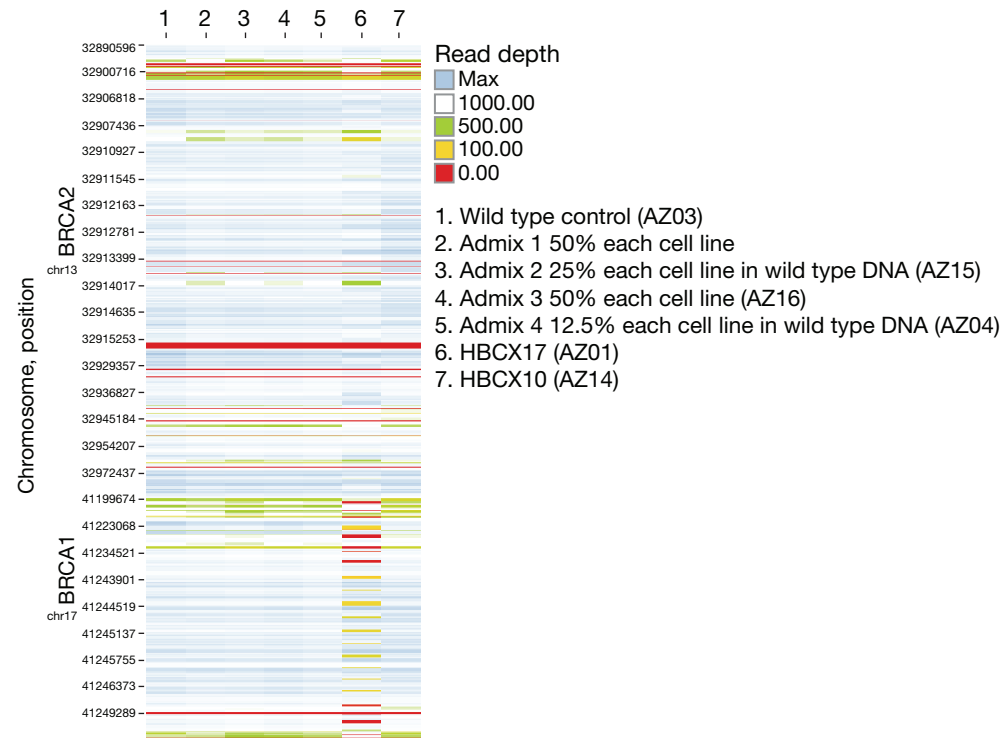

Supplement: Additional file 3: — GeneRead V.1 panel read depth on control cell lines and tumour explant samples. A mean read depth of >3,900 was obtained for all samples. Regions of lower coverage are apparent in AZ01 (column 6, fixed explant) by the red and green horizontal lines not present in the other samples. The thick red bar near the centre of the heatmap indicates a region in the BRCA2 CDS not covered in the panel design. [file 12907_2015_4_MOESM3_ESM.pdf]

Additional File 5

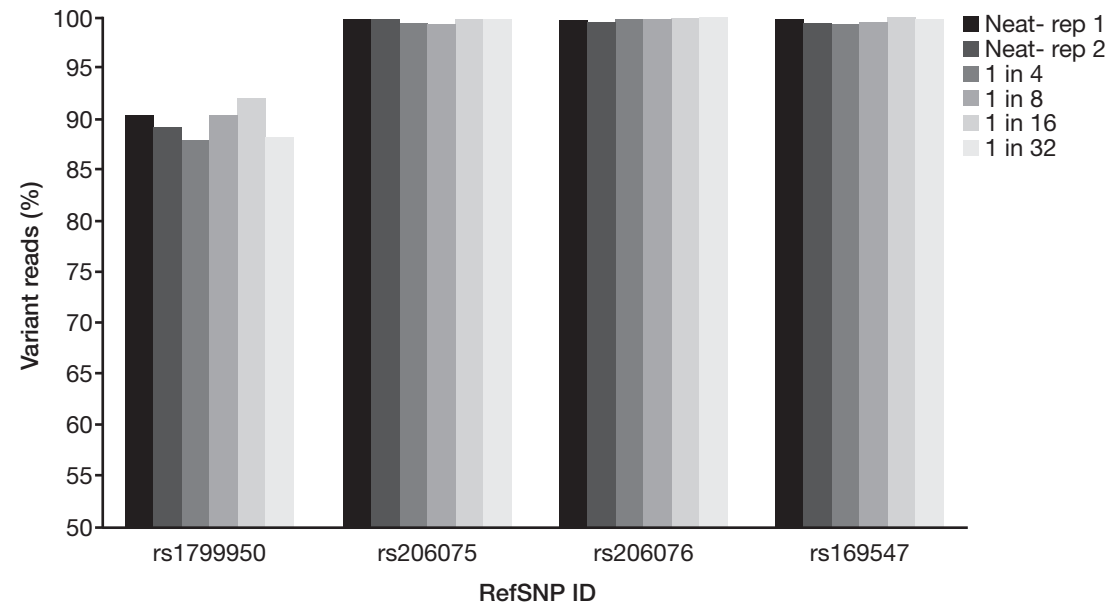

Supplement: Additional file 5: — Consistency of percentage variant reads of SNPs. The figure shows the effect on the percentage of variant reads relative to DNA input. The percentage reads remain consistent across the four SNPs in the regions of interest. The percentage read for rs1799950 is lower, but consistently so. [file 12907_2015_4_MOESM5_ESM.pdf]
