# Supplementary material for: A reliable method for the detection of BRCA1 and BRCA2 mutations in fixed tumour tissue utilising multiplex PCR-based targeted next generation sequencing
Source: BMC Clin Pathol. 2015 Mar 24;15:5. doi: 10.1186/s12907-015-0004-6 (PMC4391122; doi:10.1186/s12907-015-0004-6)
Supplement: Additional file 4: — Effect on coverage and ability to detect variants compared with DNA input. The coverage and percentage reads remained consistent as DNA input was reduced. The BRCA2 c.10095delinsGAATTATATCT p.(Ser3366AsnfsTer4) variant was still detectable at 2.5 ng total DNA input or 0.6 ng DNA input per primer pool. [file 12907_2015_4_MOESM4_ESM.doc]

**Additional file 4: Effect on coverage and ability to detect variants compared with DNA input. The coverage and percentage reads remained consistent as DNA input was reduced. The *BRCA2* c.10095delinsGAATTATATCT p.(Ser3366AsnfsTer4) variant was still detectable at 2.5ng total DNA input or 0.6ng DNA input per primer pool.**

| Sample (Variant) | Total input DNA  (ng at 129bp) | % variant reads |
| --- | --- | --- |
| AZ10 *BRCA2* c.10095delinsGAATTATATCT p.(Ser3366AsnfsTer4) | 80 | 92.9% |
|  | 20 | 91.9% |
|  | 10 | 91.7% |
|  | 5 | 92.0% |
|  | 2.5 | 93.1% |
