# Supplementary material for: A reliable method for the detection of BRCA1 and BRCA2 mutations in fixed tumour tissue utilising multiplex PCR-based targeted next generation sequencing
Source: BMC Clin Pathol. 2015 Mar 24;15:5. doi: 10.1186/s12907-015-0004-6 (PMC4391122; doi:10.1186/s12907-015-0004-6)
Supplement: Additional file 6: — Comparison of GeneRead V.1 and V.2 coverage at 100x minimum depth and mean read depth. An overall improvement in coverage and depth was observed with V.2, although the maximum coverage by design (100%) was not achieved. [file 12907_2015_4_MOESM6_ESM.doc]

**Additional file 6: Comparison of GeneRead v1 and v2 coverage at 100x minimum depth and mean read depth. An overall improvement in coverage and depth was observed with V.2, although the maximum coverage by design (100%) was not achieved.**

| Sample ID | Total input DNA  (ng at 129bp) | GeneRead v1 coverage  at a minimum 100x | GeneRead v1  Mean read depth | GeneRead v2 coverage at a minimum 100x | GeneRead v2 Mean read depth |
| --- | --- | --- | --- | --- | --- |
| AZ26 | 80 | 97.0% | 4860 | 98.4% | 9151 |
| AZ27 | 80 | 97.0% | 6450 | 98.1% | 5904 |
| AZ36 | 80 | 97.0% | 7455 | 98.1% | 6927 |
| AZ68 | 80 | 96.9% | 6417 | 98.5% | 7095 |
| AZ74 | 33.8 | 96.9% | 7468 | 97.9% | 8891 |
| AZ76 | 11.8 | 96.9% | 6249 | 97.8% | 8352 |
| AZ80 | 80 | 96.9% | 6679 | 97.9% | 12944 |
| AZ81 | 80 | 96.9% | 4341 | 97.7% | 7088 |
| AZ83 | 5.1 | 96.8 | 8519 | 96.7% | 6271 |
| AZ84 | 80 | 97.0% | 6135 | 98.1% | 6252 |
| AZ85 | 80 | 97.0% | 7327 | 98.2% | 7457 |
| AZ86 | 80 | 97.0% | 7590 | 98.9% | 9560 |
