# Supplementary material for: A reliable method for the detection of BRCA1 and BRCA2 mutations in fixed tumour tissue utilising multiplex PCR-based targeted next generation sequencing
Source: BMC Clin Pathol. 2015 Mar 24;15:5. doi: 10.1186/s12907-015-0004-6 (PMC4391122; doi:10.1186/s12907-015-0004-6)
Supplement: Additional file 7: — Coverage at 100x minimum read depth and effect on coverage at 25 and 30 PCR cycles. Coverage was better at 25 cycles for the majority of samples compared with 30 cycles. [file 12907_2015_4_MOESM7_ESM.doc]

**Additional file 7: Coverage at 100x minimum read depth and effect on coverage at 25 and 30 PCR cycles.** Coverage was better at 25 cycles for the majority of samples compared with 30 cycles.

| Sample |  | 25 cycles | | 30 cycles | |
| --- | --- | --- | --- | --- | --- |
|  | Total input DNA Ion AmpliSeq  (ng at 129bp) | Coverage at min 100x | Mean coverage | Coverage at min 100x | Mean coverage |
| AZ10 | 30 | 99.2% | 7412 | 98.9% | 9399 |
| AZ26 | 30 | 99.2% | 6064 | 98.6% | 6810 |
| AZ27 | 30 | 99.3% | 7628 | 97.1% | 11582 |
| AZ28 | 28.8 | 99.3% | 9236 | 97.6% | 8207 |
| AZ30 | 27.4 | 99.3% | 5511 | 96.1% | 15961 |
| AZ34 | 30 | 99.4% | 4706 | 94.9% | 7698 |
| AZ35 | 30 | 99.3% | 6162 | 76.6% | 8555 |
| AZ36 | 30 | 99.2% | 5733 | 94.6% | 8199 |
| AZ38 | 30 | 99.3% | 8716 | 98.5% | 6190 |
| AZ43 | 30 | 96.7% | 7125 | 98.6% | 6382 |
| AZ46 | 30 | 97.9% | 5933 | 98.6% | 11331 |
| AZ58 | 30 | 80.5% | 1416 | 96.1% | 10913 |
