# Supplementary material for: A reliable method for the detection of BRCA1 and BRCA2 mutations in fixed tumour tissue utilising multiplex PCR-based targeted next generation sequencing
Source: BMC Clin Pathol. 2015 Mar 24;15:5. doi: 10.1186/s12907-015-0004-6 (PMC4391122; doi:10.1186/s12907-015-0004-6)
Supplement: Additional file 8: — Summary of samples used in each method. [file 12907_2015_4_MOESM8_ESM.doc]

**Additional file 8: Summary of samples used in each method**

| **Sample** | **Sample type** | **High Impact Variant (confirmed)** | **Methods used** | | | |
| --- | --- | --- | --- | --- | --- | --- |
|  |  |  | **Gene**  **Read v1**  (replicate result) | **Sanger** | **Gene**  **Read v2** | **Ampli**  **Seq** |
| AZ2 | Ovary |  | X |  |  |  |
| AZ5 | Ovary |  | X |  |  |  |
| AZ7 | Ovary |  | X (2 --) |  |  |  |
| AZ8 | Ovary |  | X |  |  |  |
| AZ9 | Ovary |  | X (2 --) |  |  |  |
| AZ10 | Ovary | *BRCA2* c.10095delinsGAATTATATCT p.(Ser3366AsnfsTer4) | X (2++) | X |  | X |
| AZ11 | Ovary | *BRCA1* c.181T>G p.(Cys61Gly) | X (2 ++) | X (fail) |  |  |
| AZ12 | Ovary |  | X |  |  |  |
| AZ17 | Ovary | *BRCA1* c.2060A>C p.(Gln687Pro) | X (2 ++) | X |  |  |
| AZ18 | Ovary |  | X |  |  |  |
| AZ19 | Ovary |  | X |  |  |  |
| AZ20 | Ovary |  | X |  |  |  |
| AZ21 | Ovary |  | X (2 --) |  |  |  |
| AZ23 | Ovary | *BRCA2* c.7007+1G>C | X (2 ++) | X |  |  |
| AZ25 | Ovary |  | X |  |  |  |
| AZ26 | Ovary |  | X |  | X | X |
| AZ27 | Ovary |  | X |  | X | X |
| AZ28 | Ovary | *BRCA1* c.5266dupC p.(Gln1756ProfsTer) | X (2 ++) | X |  | X |
| AZ29 | Ovary | *BRCA2* c.9302T>C p.(Leu3101Pro) | X (+,failed replicate) | X |  |  |
| AZ30 | Ovary | *BRCA1* c.4675G>A p.(Glu1559Lys) | X (2 ++) | X |  | X |
| AZ31 | Ovary |  | X (3 +--) |  |  |  |
| AZ32 | Ovary |  | X |  |  |  |
| AZ33 | Ovary |  | X |  |  |  |
| AZ34 | Ovary |  | X |  |  | X |
| AZ35 | Ovary |  | X |  |  | X |
| AZ36 | Ovary |  | X |  | X | X |
| AZ37 | Ovary |  | X |  |  |  |
| AZ38 | Ovary |  | X |  |  | X |
| AZ39 | Ovary | *BRCA2* c.7788delAinsGGGT p.(Gly2596dup) | X (2 ++) | X |  |  |
| AZ40 | Ovary |  | X |  |  |  |
| AZ41 | Ovary |  | X (3 +--) |  |  |  |
| AZ42 | Ovary |  | X |  |  |  |
| AZ43 | Ovary |  | X |  |  | X |
| AZ44 | Ovary |  | X |  |  |  |
| AZ45 | Ovary |  | X |  |  |  |
| AZ46 | Ovary |  | X |  |  | X |
| AZ46 | Ovary |  | X |  |  |  |
| AZ48 | Ovary |  | X |  |  |  |
| AZ49 | Ovary |  | X |  |  |  |
| AZ50 | Ovary |  | X |  |  |  |
| AZ54 | Ovary |  | X |  |  |  |
| AZ55 | Ovary |  | X |  |  |  |
| AZ56 | Ovary |  | X |  |  |  |
| AZ57 | Ovary |  | X (3 +--) |  |  |  |
| AZ58 | Ovary |  | X |  |  | X |
| AZ62 | Ovary |  | X |  |  |  |
| AZ63 | Ovary |  | X |  |  |  |
| AZ64 | Ovary |  | X |  |  |  |
| AZ65 | Ovary |  | X |  |  |  |
| AZ67 | Ovary |  | X |  |  |  |
| AZ68 | Ovary | *BRCA1* c.1105delG p.(Asp369MetfsTer5) | X (2++) | X | X |  |
| AZ69 | Ovary |  | X |  |  |  |
| AZ70 | Ovary |  | X |  |  |  |
| AZ71 | Ovary |  | X |  |  |  |
| AZ72 | Ovary | *BRCA2* c.10024G>A p.(Glu3342Lys) | X (2++) | X |  |  |
| AZ73 | Ovary |  | X (3 +--) |  |  |  |
| AZ74 | Ovary |  | X |  | X |  |
| AZ75 | Ovary | *BRCA1* c.1105delG p.(Asp369MetfsTer5) | X (2 ++) | X |  |  |
| AZ76 | Ovary |  | X |  | X |  |
| AZ77 | Ovary |  | X |  |  |  |
| AZ78 | Ovary | *BRCA2* c.1408G>C p.(Glu470Gln) | X (2 ++) | X |  |  |
| AZ79 | Ovary |  | X |  |  |  |
| AZ80 | Ovary |  | X |  | X |  |
| AZ81 | Ovary |  | X |  | X |  |
| AZ82 | Ovary |  | X |  |  |  |
| AZ83 | Ovary |  | X |  | X |  |
| AZ84 | Ovary |  | X |  | X |  |
| AZ85 | Ovary |  | X |  | X |  |
| AZ86 | Ovary |  | X |  | X |  |
| AZ88 | breast |  | X |  |  |  |
| AZ89 | breast |  | X |  |  |  |
| AZ90 | breast |  | X |  |  |  |
| AZ91 | breast |  | X |  |  |  |
| AZ92 | breast |  | X (3 +--) |  |  |  |
| AZ93 | breast |  | X |  |  |  |
| AZ94 | breast |  | X |  |  |  |
| AZ95 | breast |  | X |  |  |  |
| AZ96 | breast |  | X |  |  |  |
| AZ97 | breast |  | X |  |  |  |
| AZ98 | breast |  | X (3 +--) |  |  |  |
| AZ99 | breast |  | X |  |  |  |
| AZ100 | breast |  | X (3 +--) |  |  |  |
| AZ101 | breast |  | X |  |  |  |
| AZ102 | breast |  | X |  |  |  |
| AZ103 | breast |  | X |  |  |  |
| AZ104 | breast |  | X |  |  |  |
| AZ105 | breast |  | X |  |  |  |
| AZ106 | breast |  | X |  |  |  |
| AZ107 | breast |  | X |  |  |  |
| AZ108 | breast |  | X |  |  |  |
| AZ109 | Breast | *BRCA1* c.5095C>T p.(Arg1699Trp) | X (2 ++) | X |  |  |
| AZ110 | breast |  | X |  |  |  |
| AZ111 | breast |  | X |  |  |  |
| AZ112 | breast |  | X |  |  |  |
| AZ113 | Breast | *BRCA1* c.2253_2254delGT p.(Met751IlefsTer10) | X (2 ++) | X |  |  |
| AZ114 | breast |  | X |  |  |  |
| AZ115 | breast |  | X |  |  |  |
| AZ116 | breast |  | X |  |  |  |

X: samples tested, +: significant variant identified in replicate, - no significant variant identified in replicate
